# BY4741 cannot enter quiescence from rich medium

**DOI:** 10.17912/micropub.biology.000742

**Published:** 2023-03-17

**Authors:** Shawna Miles, Cameron Lee, Linda Breeden

**Affiliations:** 1 Basic Science, Fred Hutchinson Cancer Center, Seattle, Washington, United States; 2 Tune Therapeutics, 1930 Boren Ave., Seattle, Washington, United States

## Abstract

In rich medium, W303 *Saccharomyces cerevisiae* begins to accumulate in G1 an hour before it exhausts the available glucose. It undergoes one more asymmetrical cell division, then stops dividing in G1. In contrast, BY4741, stops dividing four hours before glucose exhaustion, at one-fourth the cell density achieved by W303. There is no asymmetrical cell division and only 50% of the cells arrest in G1. We conclude that BY4741 growth is not limited by glucose and they do not go through the stereotypical events carried out by other strains as they enter quiescence from rich medium. In W303, the timing of glucose limitation and the transition to quiescence is correlated with the rate of biomass accumulation and cell doubling time.

**Figure 1. BY4741 stops dividing before the diauxic shift (DS) and fails to undergo the last asymmetric division and fails to fully arrest in G1 during the transition to quiescence in rich medium:**
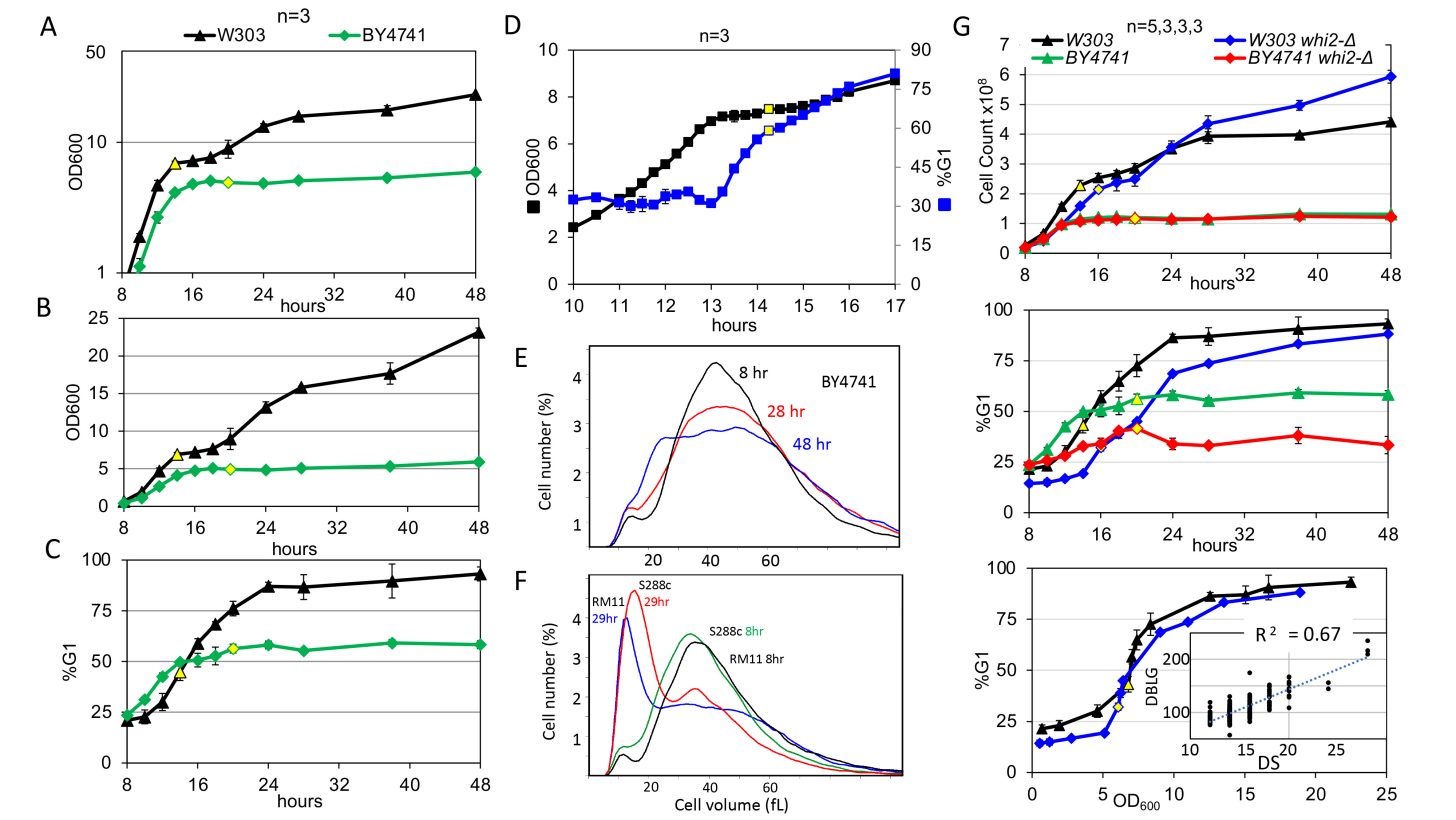
The diauxic shift (DS), which we operationally define as the time point at which all the glucose has been exhausted from the medium, is shown as yellow-filled time points. (A and B) Log and linear plots of optical density of W303 and BY4741 as a function of time. (C) Percent of cells in G1 for the same cultures. (D) Optical density (black squares) and the percent of cells in G1 (blue squares) of W303 measured at 15 minute intervals to better define when cells begin to accumulate in G1 with respect to the DS (yellow filled symbols). (E) BY4741 does not undergo an asymmetrical cell division, but (F) lab strain S288c and wild strain RM11 carry out this asymmetrical division just like W303. (G) The *whi2-Δ* phenotype differs in W303 and BY4741 in terms of cell number increase with time and percent of cells in G1 as a function of time and OD _600_ . The inset in the bottom panel shows the direct correlation between cell doubling time (DBLG) in log phase in minutes and the hour at which the DS occurs. Note that elimination of glucose from the medium is measured every two hours from 8 to 20 hours, so that limits the resolution of this experiment.

## Description

W303 and BY4741 are two of the most commonly used lab strains of *Saccharomyces cerevisiae* . The latter is particularly valuable for all the resources that have been engineered into it. Deletion libraries and tagged protein libraries have been built into BY4741 and thousands of genetic and biochemical screens have been carried out in this strain. We have been investigating the transition from proliferation to quiescence that is triggered by growth to saturation due to limited glucose in lab and wild strains, including a W303 prototroph and the BY4741 auxotroph. Prototrophs make all the amino acids and other compounds that they require as long as they have a source of essential nutrients (carbon, nitrogen, phosphate etc.) Hence, they do not experience limitations for these compounds, and these limitations have been shown to reduce longevity (Henry 1973; Saldanha *et al.* 2004; Boer *et al.* 2008) and cannot be corrected by over-supplementation (Enriquez-Hesles *et al.* 2020; Santos *et al.* 2021). Maximum growth of BY4741 in minimal medium requires supplementation for four known auxotrophies and further supplementation with phenylalanine, serine, threonine, and glutamine (Hanscho *et al.* 2012). This growth limitation is also evident in rich medium (see below).

W303 prototrophs grown in rich medium begin to slow growth and accumulate in G1 before they exhaust all the glucose from the medium and undergo the diauxic shift (DS). They undergo one more asymmetrical division and stop dividing with 95% of the cells in G1 about 24 hours later. These cells reach an optical density (OD _600_ ) of over 20 at saturation (Figure 1A and B). However, BY4741 achieves an OD _600_ and cell number of about one-quarter that attained by W303 prototrophs after 48 hours of growth. The log plot in Figure 1A shows that BY4741 displays an initial logarithmic phase of growth, but little growth takes place after 16 hours (Figure 1B). The DS (yellow symbol) for BY4741 occurs at 20 hours, which is four hours after it ceases mass accumulation, and six hours later than that of W303. The initial increase in 1N DNA from 25% to 50% takes place before the DS, but there is almost no further increase in optical density or G1 DNA content thereafter (Figure 1C). In contrast, W303 undergoes the initial increase to 50% 1N DNA one hour before the DS (Figure 1D), and then reaches 95% about 24 hours later (Figure 1C.) The cell number (p=10 ^-5^ ) and percent of cells in G1 (p=10 ^-4^ ) is significantly lower in BY4741 than in W303 after 48 hours. In addition, the asymmetric cell division that takes place in W303 after the DS and gives rise to small daughter cells with a modal cell volume of less than 20 femtoliters (Li *et al.* 2013) does not occur in BY4741 (Figure 1E.) Out of concern that our BY4741 might not be representative, we also characterized BY4741 from two other sources. All three strains behaved identically in terms of final optical density, and failure to G1 arrest. We also looked at two other yeast strains: the progenitor of BY4741 (S288c) and a wild strain (RM11), and found that both strains undergo an asymmetrical division comparable to W303 (Figure 1F).

To see how the differences between BY4741 and W303 would affect the phenotype of mutants with known defects in G1 arrest, we analyzed *whi2* in both backgrounds. *whi2* mutants were originally shown to be small cells that undergo additional divisions as they enter stationary phase and arrest randomly (Carter and Sudbery 1980; Sudbery *et al.* 1980). Contrary to expectation, *whi2* did not produce more cells during the first 48 hours in BY4741 (Figure 1G, top panel.) However, *whi2* cells were more defective in G1 arrest than BY4741 (middle panel, p=.0008.) In W303, *whi2* is small in log phase and goes through extra cell divisions as previously observed (p=.0005 at 48 hr). The *whi2* DS is two hours later than the wild type and G1 arrest is also delayed as a function of time (p=10 ^-5^ at 24 hrs). However, when plotted as a function of optical density, *whi2* behaves remarkably like wild type W303 (Figure G, bottom panel.) These data suggest that G1 arrest in W303 *whi2* is not impaired, these cells just undergo the DS later and get the signal to initiate the arrest at a later time. Whi2 has recently been shown to inhibit TORC1 (Target of rapamycin complex 1), to stop growth, and to prevent cell death in response to low amino acid levels, but not in response to low glucose (Cheng *et al.* 2008; Teng *et al.* 2018). This may explain why *WHI2* is not required for the glucose-limited, G1 arrest in W303 prototrophs, but is required for G1 arrest in the amino acid deficient BY4741.

*whi2* undergoes G1 arrest at about the same optical density that W303 arrests, but lags by up to 6 hours as a function of time. Optical density is a measure of the light scattering of the population. It reflects the total biomass of the culture and is influenced by cell size, cell number and cell wall composition (Li *et al.* 2013). Using our standardized assay, the DS occurs after 14 hours of growth at which point W303 has an OD _600_ of 6.59 ± .29 (n=37.) Using the same assay, we have followed the transition from growth to quiescence with over one hundred mutants that either have a role in G1 control during rapid growth or have been implicated in the transition to quiescence or stress response. In this mutant collection, the DS varies between 12 and 28 hours after inoculation, but the optical density at which it occurs is remarkably close to wild type (6.53 ± .46.) A simple interpretation is that glucose import is limited by the total area of cytoplasmic membrane (Phillips and Milo 2009). Consistent with this, the scatter plot in the bottom panel of Figure 1G shows that there is a direct correlation between cell doubling time during log phase and the time at which the DS occurs (R ^2^ = 0.672) in W303 and these derived strains. This variable should be taken into account when assessing delays and defects in the transition to quiescence. Size in log phase does not make a significant contribution (R ^2^ =0.013) in this data set.

We have observed large differences in the transition to quiescence in two “wild type” lab strains grown in rich medium. However, there are no publicly available, fully sequenced genomes for either of these two related strains to facilitate further understanding of these differences. BY4741 is clearly not limited for glucose when it stops dividing in rich medium and it doesn’t undergo the asymmetric cell division or the G1 arrest typical of other strains. Despite its undeniable importance in studies of log phase growth, BY4741 is not ideal for studies of the transition to quiescence. The W303 prototroph behaves more like wild strains, but it has a truncated *SSD1* allele that interferes with quiescence in haploids (Li *et al.* 2009) and kills diploids undergoing that transition (Breeden and Miles 2022). Both of these strains are mutts in the sense that they are the products of undefined crosses and neither provides an opportunity to study an optimal quiescence as it has naturally evolved. NCYC 3469 is an example of a wild strain found in fermenting fruit juice that does not sporulate, but can efficiently enter a stress tolerant, and long lived quiescent state (Miles *et al.* 2019). This and other wild strains with similar characteristics may help define the essential hardware and mechanics of the transition to quiescence (Breeden and Tsukiyama 2022).

## Methods

*Yeast Strains and Growth Conditions*

BY6500 is the prototrophic version of W303 (Li *et al.* 2009). *WHI2* in W303 was deleted using pFA6a- *KanMX* (Longtine *et al.* 1998). BY4196 is our lab stock of BY4741 obtained from the deletion consortium at Rosetta. SBY6123 and SBY19883 are two different sources of the BY4741 strain that were obtained from Dr Sue Biggins and used for comparison to our lab BY4741 strain. DMA530 is the *whi2* deletion strain from the DMA collection (Winzeler *et al.* 1999). The prototrophic RM11 and S288C were gifts from Dr Wenying Shou. Growth curve cultures were grown as described in (Miles *et al.* 2016). The YEPD rich medium used contains 10 g yeast extract, 20 g bactopeptone, 55 mg adenine hemisulfate and 2% glucose per liter.

*Cell Processing*

Flow cytometry was processed as stated in (Li *et al.* 2013). The percent G1 was calculated as described in (Miles *et al.* 2016). Cell size and number was measured with a Z2 Beckman Coulter Counter (Beckman Coulter, Brea, CA). Doubling time was calculated from the cell number increase during log phase when the cultures were at optical densities between 0.7 and 2.0.

## Reagents

**Table:** 

BY6500	*MATa can1-100 rad5 ssd1-d (W303)*
BY7288	*MATa can1-100 rad5 ssd1-d whi2Δ::KanMX*
BY4196	*MATa his3Δ1 leu2Δ0 met15Δ0 ura3Δ0 (BY4741)*
DMA530	*MATa his3Δ1 leu2Δ0 met15Δ0 ura3Δ0 whi2Δ::KanMX*
BY6941	*MATalpha RM11*
BY6942	*MATa S288c*
